# Multimodal Non-Surgical Treatments of Aggressive Pituitary Tumors

**DOI:** 10.3389/fendo.2021.624686

**Published:** 2021-03-26

**Authors:** Tae Nakano-Tateno, Kheng Joe Lau, Justin Wang, Cailin McMahon, Yasuhiko Kawakami, Toru Tateno, Takako Araki

**Affiliations:** ^1^ Division of Endocrinology and Metabolism, Department of Medicine, University of Alberta, Edmonton, AB, Canada; ^2^ Division of Diabetes, Endocrinology and Metabolism, Department of Medicine, University of Minnesota, Minneapolis, MN, United States; ^3^ Department of Genetics, Cell Biology, and Development, University of Minnesota, Minneapolis, MN, United States; ^4^ Stem Cell Institute, University of Minnesota, Minneapolis, MN, United States

**Keywords:** non-surgical therapy, Temozolomide, CAPTEM, PRRT (Peptide Receptor Radionuclide Therapy), aggressive pituitary tumors, pituitary carcinomas

## Abstract

Up to 35% of aggressive pituitary tumors recur and significantly affect mortality and quality of life. Management can be challenging and often requires multimodal treatment. Current treatment options, including surgery, conventional medical therapies such as dopamine agonists, somatostatin receptor agonists and radiotherapy, often fail to inhibit pituitary tumor growth. Recently, anti-tumor effects of chemotherapeutic drugs such as Temozolomide, Capecitabine, and Everolimus, as well as peptide receptor radionuclide therapy on aggressive pituitary tumors have been increasingly investigated and yield mixed, although sometimes promising, outcomes. The purpose of this review is to provide thorough information on non-surgical medical therapies and their efficacies and used protocols for aggressive pituitary adenomas from pre-clinical level to clinical use.

## Introduction

Pituitary tumors are mostly benign and progress slowly. Most of them are non-invasive and cured by surgery or controlled by long-term pharmacologic treatment. However, some pituitary tumors exhibit continued growth despite conventional therapies, including multiple surgeries, radiotherapy, and medical treatment (1). Recent evidence suggests that Temozolomide (TMZ), an alkylating agent, can be used as a valuable first-line chemotherapy for treatment of aggressive pituitary tumors and carcinomas (1). However, some patients may not respond to TMZ. Moreover, many patients experience disease progression or disease recurrence after completion of TMZ treatment, although tumors and carcinomas regressed during the treatment (1–13). In order to ensure long-term positive outcomes in cases of aggressive pituitary tumors, alternative treatment options are needed. Recent case reports showed that TMZ in combination with capecitabine had an improved progression free survival in aggressive pituitary tumors (14, 15). Moreover, several new therapies have been reported. These novel therapies include peptide receptor radionuclide therapy (PRRT) (16–24) and treatment with mTOR inhibitors (11, 25–27), epidermal growth factor receptor (EGFR) inhibitors (28), immune checkpoint inhibitors (29–31), cyclin dependent kinase (Cdk) inhibitors (32), or vitamin A derivatives (33, 34). In this review, we describe the mechanisms, efficacies, and protocols of each treatment and summarize the studies and cases published in the literature.

## Temozolomide (TMZ)

TMZ is an orally-administered alkylating agent that was originally used for treatment of glioblastomas combined with radiation therapy (35). TMZ was first used in 2006 to manage high-risk pituitary tumors (36–38). TMZ induces methylation of guanine residue at the O6 position in the DNA. O6-methylguanine incorrectly pairs with thymine and triggers the mismatch repair system, leading to the formation of double-strand breaks in the genome, which causes cell cycle arrest and induction of apoptosis (39).

**Figure 1:**
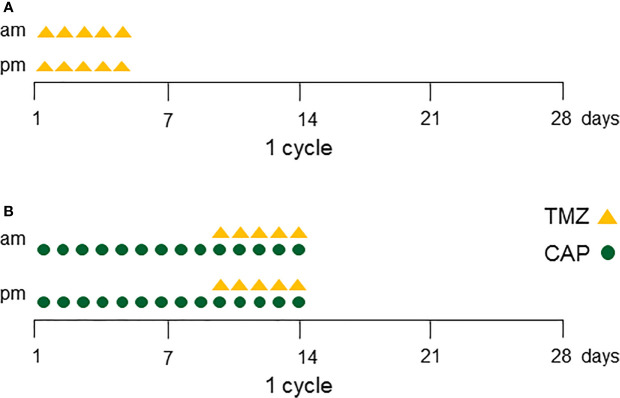
**(A)** One cycle of Temozolomide (TMZ) protocol. TMZ is given twice daily (150–200 mg/m^2^/day) for 5 consecutive days every 28 days (=1 cycle). After three cycles of TMZ, MRI is taken for treatment evaluation. Continue TMZ at least six cycles in total for responder patients. **(B)** One cycle of Capecitabine (pro-drug of 5-Fluorouracil) + Temozolomide (CAPTEM) protocol. Oral capecitabine (CAP) is given (1,500 mg/m^2^/day) on days 1 through 14 divided into two doses, and TMZ is given twice daily (150 to 200 mg/m^2^/day) on days 10 through 14. This 2-week regimen is followed by 2 weeks off treatment. Continue CAPTEM at least 12 cycles in total for responder patients.

TMZ was recommended as first-line chemotherapy for aggressive pituitary tumors and carcinomas by the European Society of Endocrinology in 2018 (1) and usually elicits an immediate response. The standard dose of TMZ is 150–200 mg/m^2^ for 5 consecutive days every 28 days (=1 cycle) for at least six cycles (1) (**Figure 1A**). TMZ is a generally well-tolerated therapy. The major side effects are fatigue and nausea. Twelve studies with greater than five patients have been published since the first case of TMZ use in the management of aggressive pituitary tumors in 2006 (2–13) (**Table 1**). The total cumulative number of patients is 366, including 113 with pituitary carcinomas. Although follow-up duration and definition of a response differed among studies, the overall response in the reduction of tumor size ranged from 33%–87%. In the largest study by the European Society of Endocrinology, 166 patients were treated with TMZ as first-line chemotherapy. 37% of the patients showed radiological response and 33% showed stable disease (11). Similarly, the second largest study with 47 patients, including 13 with pituitary carcinomas, reported that 37% of patients exhibited tumor size reduction following TMZ treatment (13). In a meta-analysis of 106 patients from 11 studies of aggressive pituitary tumors, 47% of the patients showed reduction in tumor size (1).

**Table 1:** Temozolomide (TMZ) treatment response in pituitary tumors and carcinomas from published case series of 5 or more patients.

response in tumor growth with medication	TMZ (cycles) follow-up(months)	follow-up (months)	recurrent rate (%) and occurred timing after treatment (month)	MGMT correlations	case numbers (cancer)	ref, year [ref no]
CR/PR;51%	2–24	16	46%; after 5 months	No	43 (14)	Lasolle (2)
PR/CR;29%, SD;58%, PD14%	2–13	ns	ns	No	7 (2)	Bush (3)
CR/PR/SD; 38%	3–24	ns	ns	No	8 (5)	Raverot (4)
CR/PR;33%, SD;33%, PD;33%	3–12	22.5	33%; after 6 months	Yes	6 (1)	Losa (5)
CR/PR;31%, SD;15%, PD;54%	3–24	ns	46%; after 10.5 months	No	13 (10)	Hirohata (6)
CR/PR;33%	3–6	ns	ns	ns	6 (1)	Bruno (7)
CR/PR;43%	1–23	32.5	33%	Yes	21 (8)	Bengtsson (8)
PR;40%, SD;20%, PD;40%	3–24	ns	ns	ns	5 (0)	Ceccato (9)
CR/PR;36%, SD;45%, PD;19%	3–12	43	52%; after cessation	No	31 (6)	Losa (10)
CR;6%, PR;31%, SD;33%, PD;30%	1–36	21	38%; after 12 months	Yes	166 (40)	McCormack (11)
TMZ-based therapy was associated with the longest PFS (71%) and long disease control	−12	28	62%	ns	13 (13)	Santos (12)
CR/PR;20%, SD;17%, PD;63%	1-26	32	63%; after 16 months	No	47 (13)	Elbelt (13)

ns, not stated; CR complete response; PR, partial response; SD, stable disease; PD, progressive disease; PFS, progression free survival.

TMZ treatment outcome differs between hormone secreting and non-hormone secreting pituitary tumors. A review of 8 case series encompassing 100 pituitary tumor cases treated with TMZ showed that corticotroph adenomas and prolactinomas have about a 56% and 44% tumor reduction rate, respectively. In contrast, non-functioning pituitary adenomas have only a 22% of tumor response rate (40). Two other studies also showed that patients with functioning tumors exhibited better tumor size reduction after treatment with TMZ compared to those with NFPAs (2, 11).

As mentioned above, immediate responses to TMZ are mostly favorable; however, tumor progression and relapse frequently occur during long-term follow-up (33%–63%) (**Table 1**). In two large-scale studies with long-term follow-up, tumor relapse occurred in 46% (16 month follow up with 5 months after cessation) and 38% (21 months follow-up with 12 months after cessation) of patients, respectively (2, 11). In another study that focused on long-term effects of TMZ, up to 63% of the patients demonstrated disease progression after a median of 16 months following initiation of TMZ (13). Longer TMZ treatment duration (more than 12 cycles) was found to be associated with better survival free of tumor-progression (1, 2, 11, 41). A retrospective analysis showed higher survival rate in the long-term treatment group (longer than 12 months) compared to the short-term (1–12 months) treatment group [5-year overall survival 92% vs 54% (41)], suggesting that longer treatment cycles can increase the likelihood of sustained remission. However, reports from many cases showed that a second course of TMZ failed to induce tumor regression if tumor progression or systemic metastases occurred after an initial treatment (2, 8, 10, 42, 43). Therefore, re-challenging with TMZ is not recommended if an initial treatment fails to control disease progression. One of the reasons for such a relapse might be related to TMZ-induced hypermutations in genes such as *MSH6, CDKN2A/B*, and *PIK3CA*, which can be associated with TMZ resistance (29).

Several biomarkers that may predict response to TMZ have been identified. One such marker is O6-methylguanine DNA methyltransferase (MGMT), a DNA-repair enzyme, which counteracts TMZ-induced DNA damage (44). The expression of MGMT seems to correlate with response of a tumor to the TMZ therapy. A number of studies indicate an association between low expression levels of MGMT (determined by immunostaining) and better treatment response to TMZ (1, 5, 8, 11, 45–51). However, this association was not observed in several other studies (2–4, 6, 10, 13) (**Table 1**). This discrepancy could be, at least in part, derived from a lack of standardized scoring systems for MGMT immunostaining. In addition, the expression level of MGMT may increase during TMZ therapy (45), which could affect interpretation of MGMT expression. At present, the expression of MGMT is still considered a predictive marker for TMZ response. Another biomarker is MutS Homolog 6 (MSH6), a DNA mismatch repair protein. Mutations in the *MSH6* gene in glioblastoma are known to be associated with TMZ resistance (52). One study showed that expression of MSH6 was positively correlated with pituitary tumor regression following treatment with TMZ (6); however, a follow-up study failed to confirm this correlation (8).

## TMZ-Based Combination Therapies: Capecitabine (Pro-Drug of 5-Fluorouracil) + Temozolomide (CAPTEM)

Capecitabine (orally administered systemic pro-drug of 5-Fluorouracil) is an antimetabolite that incorporates 5-fluorodeoxyuridine triphosphate into genomic DNA. Capecitabine causes attenuation of MGMT DNA repair activity through thymidylate synthase inhibition and reduction of the thymidine level, which enhances the apoptotic effect of TMZ (53, 54). CAPTEM is a novel combination of capecitabine and TMZ. Two *in vitro* experiments investigating anti-tumor effects of CAPTEM have been reported. One study in human carcinoid cell lines showed synergistic cytotoxicity when 5-fluorouracil (5-FU) exposure was preceded by TMZ (55). The other study, conducted by the authors of this review, used mouse corticotroph tumor cells to demonstrate that 5-FU treatment in combination with TMZ had an additive effect both in decreasing cell viability and reducing the amount of ACTH released by the tumor cells in the culture medium (15).

The clinical protocol of CAPTEM consists of oral capecitabine 1,500 mg/m^2^/day (maximum daily dose of 2,500 mg on days 1 through 14 divided into two doses) and TMZ 150 to 200 mg/m^2^/day (oral divided into two doses) given on days 10 through 14. This 2-week regimen is followed by 2 weeks off-treatment [**Figure 1B** (14)]. Data obtained from *in vitro* experiments support the efficacy of two separate TMZ treatments (56). Specifically, the first treatment causes partial reduction of MGMT activities, whereas the second treatment is responsible for the methylation of guanine residues once repair activity by MGMT has been attenuated (53).

CAPTEM is generally well tolerated; among 13 patients with aggressive pituitary tumors/carcinomas, 12 patients were able to tolerate the treatment without discontinuing therapies (**Table 2**). Three patients developed thrombocytopenia but only one of these patients had to discontinue CAPTEM due to thrombocytopenia and poor tolerance (29). Another patient developed lymphopenia without discontinuing treatment, and one case reduced to 75% of the maximal dose of CAPTEM due to nausea (15). The first reported use of CAPTEM for pituitary tumors was for an aggressive corticotroph pituitary carcinoma in 2011 (57). To date, 13 TMZ naïve cases of aggressive pituitary tumors/carcinomas treated with CAPTEM (TMZ naïve cases) have been reported, of which seven were carcinomas (7/13) and 11 were ACTH positive (11/13) (11, 14, 15, 26, 29, 57–59) (**Table 2**). Clinical or radiological improvements were observed in 11 out of 13 cases, in which tumor progression stopped for up to 54 months. In seven patients with carcinomas (five ACTH positive, one PRL positive, and one PIT-1 positive), five patients (71%) initially showed partial reduction of tumor size in response to CAPTEM treatment and no tumor progression for as long as 39 months. These reports include two cases of pituitary tumors treated with CAPTEM: one is a corticotroph carcinoma and the other is an aggressive corticotroph tumor (15). Both patients had undergone previous surgical and radiologic therapies. The first patient with corticotroph carcinoma started to receive the CAPTEM treatment after developing leptomeningeal spread. Twelve cycles of the treatment resulted in tumor control associated with improvement of clinical and radiological tumor size reduction for 39 months. The patient restarted CAPTEM after recurrence of the tumor, and is currently under treatment. The second patient, with an aggressive corticotroph tumor, was treated with 12 cycles of CAPTEM, which led to tumor shrinkage with no tumor regrowth 22 months after cessation of therapy. As for the side effects of CAPTEM, the second patient had nausea, which was manageable by reducing the dose of CAPTEM to 75% of the maximal after the 6^th^ cycle.

**Table 2:** Published cases of Capecitabine (pro-drug of 5-Fluorouracil) + Temozolomide (CAPTEM) (TMZ naïve) for aggressive pituitary tumors and carcinomas.

Response in tumor growth/PFS (month)	CAPTEM (cycles)	Tumor subtype	Pathology	Previous treatment	Side effects	Age/sex	Ref, year [ref no]
PR, PFS (22)	12	ACTH	MGMT: ns	ns	ns	44/M	McCormack (11)
PR, PFS (6)	6	ACTH	MGMT: ns	ns	ns	49/M	McCormack (11)
PD	18	PRL-CA	MGMT: ns	ns	ns	38/M	McCormack (11)
SD, PFS (54+)	30	ACTH	MGMT:low, Ki-67: <5%	TSS, BAD, RT	thrombocytopenia	50/M	Zacharia (14)
CR, PFS (32+)	32	ACTH	MGMT:low, Ki-67: 5%	TSS, RT	lymphopenia	46/F	Zacharia (14)
CR, PFS (45+)	45	Silent ACTH	MGMT:low, Ki-67: <5%	TSS, RT	none	44/M	Zacharia (14)
PR, extra-axial met	12 off 27 mon, 12 ongoing	Silent ACTH-CA	MGMT: low, Ki-67: 1-5%	TSS, RT	mild constipation 12	54/M	Nakano-Tateno (15)
PR, PFS (34+)	12	ACTH	MGMT: low, Ki-67: <1%	TSS, RT	nausea	48/M	Nakano-Tateno (15)
PD	3	PIT1-CA	MGMT: ns, Ki-67: 15%	TSS, RT	ns	23/F	Alshaikh (26)
PR for 24 mo, then liver met	4	ACTH-CA	MGMT+ (liver)	TSS, BAD, RT)	thrombocytopenia	35/F	Lin (29)
PR, PFS (5.5)	4	ACTH-CA	MGMT: ns, Ki-67:31%	TSS, BAD, RT	none	50/M	Thearle (57)
PR,spine and pelvic met	8	ACTH-CA	MGMT:ns, Ki-67:19-50%, p53+	TSS, RT	thrombocytopenia	46/F	Donovan (58)
SD, PFS (8)	8	ACTH-CA	MGMT:ns, Ki-67: 4-15%	TSS, RT	ns	49/F	Joehlin-Price (59)

ns, not stated; CR, complete response; PR, partial response; SD, stable disease; PD, progressive disease; PFS, progression free survival; CA, carcinoma TSS, transsphenoidal surgery; BAD, bilateral adrenalectomy; RT, external beam radiotherapy; mo, months; met, metastasis.

CAPTEM has been used in TMZ resistant cases as well. CAPTEM treatment followed by TMZ monotherapy (TMZ resistant cases) has been reported in eight cases (three ACTH secreting carcinomas, two PRL secreting carcinomas, one null-cell carcinoma, and two cases not stated) (8, 10–12, 42). However, the outcome was unfavorable in seven cases (88%), and only one (null- cell carcinoma) had a partial regression of carcinoma (42). From this limited dataset, CAPTEM seems to be more effective in TMZ naïve cases compared to those with TMZ resistance. Unlike TMZ monotherapy, it is unknown whether MGMT expression in tumors can be a predictive marker for response to CAPTEM. In six aggressive pituitary tumors treated with CAPTEM, five cases (83%) showed low MGMT expression levels, and one case had positive expression in the liver metastasis (14, 15, 29, 57). The outcome of these low MGMT cases varies, including cases of complete regression, partial regression, and stabilization of the tumor. Further studies are required to determine the role of MGMT expression in predicting response to CAPTEM therapy.

Other than CAPTEM, there are a few case reports of TMZ in combination with other therapeutic agents. Among them are the combinations of TMZ with VEGF-targeted therapy (Bevacizumab or Apatanib) (60–62) and a somatostatin receptor ligand (Pasireotide) (9, 63). In a survey by the European Society of Endocrinology, 1 case treated with TMZ in combination with bevacizumab achieved a partial tumor regression, 1 case with thalidomide showed no progression of the disease, and 1 case with Carmustine showed progressive disease (PD) (11). Although a prospective clinical trial is required to determine whether the treatment with CAPTEM is superior to the treatment with TMZ alone, CAPTEM appears to be a promising treatment option for aggressive pituitary tumors and carcinomas based on several case reports and our experimental data.

## Peptide Receptor Radionuclide Therapy (PRRT)

Peptide Receptor Radionuclide Therapy (PRRT) is a form of targeted therapy that utilizes the delivery of radionuclide-bound somatostatin agonists to pituitary tumors expressing somatostatin receptors (SSTRs). PRRT has successfully treated neuroendocrine tumors, due to their high levels of SSTR expression (64–66). The use of PRRT for pituitary tumors was introduced after its success in treating neuroendocrine tumors (17). Radionuclides such as Yttrium 90 (Y-90), Lutetium 177 (Lu-177), and Indium-111 (In-111) are combined with peptides or somatostatin agonists (e.g., DOTATOC, DOTATATE) to deliver radiation to tumor cells. Because this is a targeted therapy, the risk of the systemic adverse effects is lower than conventional radiation therapy (18, 38).

There is still limited evidence whether PRRT is effective in the management of aggressive pituitary adenomas or carcinomas (8, 16–24) (**Table 3**). From the review of English literature between the years of 2012 and 2020, we found a total of 15 cases describing PRRT treatment in aggressive pituitary tumors (11 cases) and carcinomas (four cases). The treatment protocols used (dose, type of radionuclide and peptide, timing of radionuclide delivery) and measured outcomes across these cases vary greatly and are summarized in **Table 3**. Broadly, regarding tumor size, among 15 reported cases, six cases (43%) responded to PRRT. Decrease in hormone levels was reported in only three cases (20%, **Table 3**).

**Table 3:** Published cases of peptide receptor radionuclide therapy (PRRT) for pituitary tumors and carcinomas.

Response in tumor growth	Hormone reduction	PFS (month)	Tumor subtype	Initial tumor volume (ml)	Total radiation dose/(number of cycles)	Type of radionuclide	Previous treatment	Age/sex	Ref, year [ref no]
n/a	ns	5	NFPA	ns	ns	177Lu-DOTATE	TMZ	59/F	Bengtsson (8)
PD	ns	8	GH-CA	ns	ns	90Y-DOTATE	TMZ	46/M	Bengtsson (8)
PD	ns	8	PRL	ns	ns	68Gallium DOTATE	TMZ	23/M	Bengtsson (8)
n/a	ns	ns	NFPA-CA	4.1ml	0.15 GBq (one dose)	177Lu-DOTATE	none	71/F	Kumar (16)
decreased 95% over 8 years	PRL decreased	ns	PRL	63 mL	37 GBq (5)	111In-DTPA-octreotide	TSS, RT, octreotide	58/F	Baldari (17)
SD over 8 years	ns	96	NFPA	ns	0.6 GBq (3)	177Lu-DOTATE	TSS	55/M	Komor (18)
Decreased 60.5% over 12 months	IGF decreased	12	GH	23.1ml	0.4 GBq (0.1 GBq every 3 mo)	90Y-DOTATE	TSS, RT, octreotide, lanreotide	26/M	Waligórska-Stachura (19)
Pituitary: SD, met volume: decrease	ns	40	NFPA-CA	ns	29.6 GBq (one dose)	177Lu-DOTATE	TSS, RT	63/M	Maclean (20)
PD	ns	ns	GH/PRL	ns	15.3 GBq (2)	177Lu-DOTATE	TSS, RT, TMZ, lanreotide	42/M	Maclean (20)
PD	ns	ns	ACTH	ns	ns (one dose)	177Lu-DOTATE	TSS, RT, TMZ	32/M	Maclean (20)
SD over 1 year, then pituitary apoplexy	GH decreased, IGF persistently high	12	GH	31.8ml	22.2 GBq (3)	177Lu-DOTATE	surgical attempt	48/M	Assadi (21)
SD over 4 years	ns	48	NFPA-CA	ns	22.2 GBq (3)	177Lu-DOTATE	TSS, RT	68/M	Novruzov (22)
PD	ns	ns	PRL	20.2 ml	12.6 GBq (2)	177Lu-DOTATOC	TSS, RT, TMZ	54/M	Giuffrida (23)
PD	ns	ns	NFPA	7.7 mL	29.8 GBq (5)	177Lu-DOTATOC	TSS, RT, TMZ	53/F	Giuffrida (23)
PD	ns	<12	ACTH-CA	ns	0.2 GBq (one dose)	90Y-DOTATOC	TSS, RT	16/F	Kovács (24)

ns, not stated; NFPA, non-functioning pituitary adenoma; CA, carcinoma; TSS, transsphenoidal surgery; RT, radiation therapy; CR, complete response; PR, partial response; SD, stable disease; PD, progressive disease; PFS, progression free survival; mo, months.

In general, PRRT treatment is tolerated well without major side effects. In a study of 30 patients with advanced cancers (mostly neuroendocrine tumors) receiving [111In-DTPA^0^] octreotide up to a high cumulative dose of 75Gbq, no major side effects were noted. Transient reduction in platelets and leukocytes can occur (67). From our review of 15 cases of aggressive pituitary tumors treated with PRRT, one patient developed transient grade 2 thrombocytopenia (20).

There is no clear evidence as to whether dosage or type of radionucleotide affects outcomes (23). From our review of 15 cases, total average radiation dose seems similar, with an average of 15.4 GBq in responders versus an average dose of 14.5 GBq in non-responders. However, dosages varied widely in both groups (from 0.4 GBq to 37 GBq), therefore it is difficult to assess whether dosage affects outcomes or not. It is also unknown whether the pre-treatment radioisotope uptake scan corresponds with treatment outcomes (18, 22, 23). In well-differentiated neuroendocrine tumors and medullary thyroid cancers, the total amount of pre-treatment radioisotope uptake did not reflect prognosis well, but heterogeneous uptake may correspond with poor prognosis after PRRT treatment (68, 69). In pituitary tumors, dosimetry analysis was reported in only 1 case, which showed a heterogeneous uptake pattern with progressed disease (20).

It is of interest to know the difference in efficacy between somatostatin analog (SSA) therapy and PRRT, however, at this point, there is no direct comparison study. At this stage, PRRT is a treatment option for aggressive pituitary tumors that express SSTR and are resistant to other therapies. The 2018 guidelines of the European Society of Endocrinology listed PRRT as an alternative treatment option for aggressive pituitary tumors (1). Taken together, current evidence does not suggest that PRRT is an impressively effective treatment for aggressive pituitary tumors, however it is still premature to conclude that PRRT is not an important treatment strategy since half of the reported cases were TMZ resistant which are usually difficult to control disease progressions.

## mTOR Inhibitors

The phosphatidylinositol 3-kinase/mammalian target of rapamycin (mTOR) pathway is involved in the regulation of survival, growth, protein synthesis, and cellular metabolism (70–73). Everolimus (EVE) is an orally active mTOR inhibitor FDA-approved to treat neuroendocrine tumors. EVE forms a complex with mTORC1, affecting downstream cellular activities, and causes cell cycle arrest and protein synthesis inhibition. Many studies have shown that EVE is effective in human pituitary tumor cultures and murine cell lines *in vitro* (74–77). It is also effective *in vivo* in intracranial GH4 cell xenograft mouse models and reduced transplanted tumor cell viability and proliferation (78). Moreover, EVE synergized with SSAs, such as octreotide or pasireotide, and exhibited anti-proliferative effects in primary human pituitary tumor cells (79, 80).

Although EVE has been widely used in the management of pancreatic and other neuroendocrine neoplasms, there are only seven published cases of pituitary tumors treated with EVE combination therapy (three ACTH secreting carcinomas, one PRL secreting adenoma, and three not stated) (11, 25–27, 58) (**Table 4**). Of note, five out of seven (71%) cases had disease progression, and tumors in all the failed cases were TMZ resistant. The protocol used for EVE treatment is variable. The reported dose of EVE is 5 to 10 mg daily for several months, either as a monotherapy or in combination with other therapies. Common side effects include stomatitis, rash, fatigue, diarrhea, infections, anemia, and hyperglycemia (25). Initial reduction in hormone secretion was observed in only one out of seven patients treated with EVE (25). A patient with a PRL secreting tumor treated with EVE 10mg + octreotide showed decreased PRL levels (454 ng/ml to 253 ng/ml; 55% reduction) after an initial 3 months of EVE therapy; however, PRL level gradually increased to its previous level by 5 months following initiation of EVE therapy (25). Among the seven published cases, only two out of seven cases (29%) reported no progression of tumor growth: one corticotroph carcinoma treated with EVE (7.5 mg) + Capecitabine followed by EVE (7.5–10 mg) + RT showed no increase in tumor volume for 5 months (58), and an aggressive PRL secreting tumor treated with EVE 10mg + octreotide showed stable tumor volume for 12 months (25). Currently, there is not a sufficient number of cases with EVE treatment for pituitary tumors to determine its effectiveness. In addition, the complexity of the reported cases may have biased the outcomes of EVE treatment. For example, three out of seven reported cases were pituitary carcinomas and six out of seven EVE treated cases were TMZ refractory cases. Further work will be required to define the role and effectiveness of EVE in management of pituitary tumors.

**Table 4:** Published cases of Everolimus (EVE) treatments for pituitary tumors and carcinomas.

Response in tumor growth	EVE treatment	Duration (month)	Tumor subtype	Pathology	Previous treatments	Age/sex	Ref, year [ref no]
PD	EVE	ns	ns	ns	TMZ	ns	McCormack (11)
PD	EVE	ns	ns	ns	TMZ	ns	McCormack (11)
PD	EVE	ns	ns	ns	TMZ	ns	McCormack (11)
tumor size: SD for 12+mon, hormone reduction: PR for 8 mon	EVE+OCT	12+	PRL	Ki-67: 30%, p53+	Surgery, RT, CAB	62/M	Zhang (25)
PD	EVE	ns	ACTH-CA	Ki-67: 10%	Surgery, BAD, RT, TMZ	49/F	Alshaikh (26)
PD	EVE+OCT	1	ACTH-CA	Ki-67: low, mitoses+	TMZ	45/M	Jouanneau (27)
SD for 5 mon then PD	EVE+paliative RT, EVE+Capecitabine	8	ACTH-CA	Ki-67: 19-50%, p53+	Surgery, BAD, RT, CAB, CAPTEM	46/F	Donovan (58)

ns, not stated; CA, carcinoma; BAD, bilateral adrenalectomy; RT, radiation therapy; SD, stable disease; PD, progressive disease.

## Immunotherapy

Immune checkpoint inhibitors upregulate the body’s immune response to fight against malignancy and have been used to treat melanomas, lung cancers, renal cancers and Hodgkin lymphomas (81). Pembrolizumab (PEM) and nivolumab (NIV) are checkpoint inhibitors that inhibit programmed cell death 1 (PD-1), which is a transmembrane protein expressed on immune cells that inhibits T-cell destruction of tumor cells. Ipilimumab (IPI) is a checkpoint inhibitor that inhibits the action of Cytotoxic T-lymphocyte-associated protein 4 (CTLA-4), which downregulates immune response (82). CTLA-4 and PD-1 are both expressed in pituitary adenomas (83–85). This is thought to be a mechanism by which immunotherapy can contribute to the treatment of pituitary tumors. However, the use of checkpoint inhibitors is associated with several adverse effects, including hypophysitis (86). For example, single-agent anti-PD-1/PD-L1 monoclonal antibody therapy is associated with incidence rate of hypophysitis between 1% to 6% (87). Combination of IPI (CTLA-4) plus NIV (PD-1) is associated with hypophysitis rate as high as 7.7% to 11.7 percent (88, 89). Other adverse effects by ICIs include thyroiditis (1%–6%) (90), primary adrenal insufficiency (0.7%) (91), dermatitis (34%–39%) (92), and hepatotoxicity (5%–10% with PD-1) (93).

Thus far, checkpoint inhibitors have been used to treat pituitary adenomas in five cases (29–31, 94) (**Table 5**). Of these five cases, three cases were ACTH-producing carcinomas, one case was an ACTH-producing adenoma, and one case was a prolactinoma. All ACTH-producing carcinoma cases received a combination of IPI and NIV and showed decreased to stable sizes of pituitary carcinomas, ACTH levels, and the liver metastatic volume in two cases (29, 30, 94). However, one of these positively responding cases of pituitary carcinoma recurred over 1 year. Of note, two other cases of non-carcinomas [ACTH-producing adenoma (treated with PEM) and prolactinoma (treated with IPI and NIV)] had disease progression despite immunotherapy (31, 94).

**Table 5:** Published cases of Immunotherapy treatments for pituitary tumors and carcinomas.

Response in tumor growth	Hormone reduction	Treatment	Tumor subtype	Pathology	Previous treatment	Age/sex	Ref, year [ref no]
intracranial: decreased by 59%, liver met: decreased by 98%. SD after 6 months	Decreased ACTH by 100%	IPI and NIV 5 cycles followed by NIV only	ACTH-CA	Liver: mitotic index 50%, PDL-1 <1%	TSS, RT BAD, TMZ	41/F	Lin (29)

SD	Decreased ACTH by 30%, am cortisol by 64%, UFC by 74%	IPI and NIV 4 cycles, then NIV maintenance and ketoconazole	ACTH-CA	Ki-67:<1%	TSS, RT, TMZ	41/M	Sol (30)
PD	PD	PEM 4 cycles	ACTH	MIB>3%, PDL-1 negative	TSS, RT, TMZ	66/M	Caccese (31)
Pituitary: decreased by 15%, liver met: decreased by 57–69%. PD after 12 months	Decreased ACTH by >93% then PD	IPI and NIV 5 cycles followed by NIV 21 cycles	ACTH-CA	Ki-67: 5%, Liver: Ki-67 10%, PDL-1 negative	TSS, RT, TMZ	60/F	Duhamel (94)
PD	PD	IPI and NIV 2 cycles	PRL	Ki-67: 25%	TSS, RT, TMZ	68/M	Duhamel (94)

NFPA, non-functioning pituitary adenoma; CA, carcinoma; TSS, transsphenoidal surgery; RT, radiation therapy; BAD, bilateral adrenalectomy; PEM, pembrolizumab; IPI, ipilimumab; NIV, nivolumab; HR, hormonal response; SD, stable disease; PD, progressive disease; UFC, urinary free cortisol.

## Conventional Therapies

### DAs Based Combination Therapies for Aggressive Prolactinomas

Although the dopamine agonists (DAs) (bromocriptine (BRC) and cabergoline (CAB)) are established first-line treatments for prolactinomas (95), DA resistance in prolactinomas occurs in 20%–30% of patients treated with BRC and 10% of patients treated with CAB (96). Possible mechanisms include decreased expression of dopamine receptor D2 (D2R), changes upstream or downstream of D2Rs signaling, increased angiogenic markers, and disruptions in the TGF- β1 pathway (97). It has been reported that higher dose of CAB therapy is successful in treating BRC resistant prolactinomas (96). Patients are generally able to tolerate high doses of DAs (98), but periodic echocardiogram may be required for prolonged high-dose use since cumulative dosage increases the risk of valvular heart disease (99). In addition to monotherapy, DA therapies in combination with tamoxifen (TAM) or octreotide (OCT) have been reported to treat DA-resistant prolactinomas with various response rates, summarized in **Table 6** (100–105). Metformin was also used in combination therapies (103). A case series with DAs and metformin over 8-14 months have noted both PRL normalization and significant tumor reduction (103).

**Table 6:** Summary of conventional therapies for aggressive prolactinomas, Cushing’s disease, and acromegaly and non-functional pituitary adenomas.

Tumor subtype	Response in tumor growth	Hormone reduction	Treatment	Duration	Case numbers	Ref, year [ref no]
PRL	38%	0%	2.5 mg/day BRC, 20 mg/day TAM	5 days	8	Lamberts (100)
	ns	58%	2.5–7.5 mg/day BRC, 10–20 mg/day TAM	4 weeks	12	Volker (101)
	100%	100%	4 mg/week CAB, 20 mg three times daily TAM	8 months	1	Christian (102)
	100%	100%	15 mg/day BRC, 1.5g/day MET	12 to 14 months	2	Liu (103)
	0%	100%	3 mg/week CAB, 20 mg/month OCT	12 months	1	Fusco (104)
	40%	0%	3–7.5 mg/week CAB, 20 mg/month OCT	6 to 13 months	5	Sosa-Eroza (105)
NFPA	32%	ns	2 mg/week CAB	6 months	19	Garcia (106)
	0%	ns	1 mg/week CAB	12 months	12	Lohmann (107)
	44%	ns	1–3 mg/week CAB	12 months	9	Pivonello (108)
	67%	ns	3 mg/week CAB	6 months	9	Vieira Neto (109)
	29%	ns	3.5 mg/week CAB	24 months	59	Batista (110)
	35%	ns	0.5–3.5 mg/week CAB or 2.5–10 mg/day BRC	ns	79	Greenman (111)
GH	ns	39%	1 to 3.5 mg/week CAB, 30 mg/28 days OCT	3 to 18 months	13	Cozzi (112)
	ns	50%	1 to 3.5 mg/week CAB, 60 mg/28 days LAN	3 to 18 months	6	Cozzi (112)
	0%	56%	1.5 or 3.5 mg/week CAB, 20–30 mg/28 days OCT	2 to 12 months	34	Jallad (113)
	ns	40%	1 to 3 mg/week CAB, 30 mg/28 days OCT	6 months	52	Vilar (114)
	11%	22%	0.25–2 mg/week CAB, 20–40 mg/month OCT	8.1 to 80.5 months	9	Suda (115)
	ns	9%	0.5–3.5 mg/week CAB, 40 mg/28 days OCT	8 months	32	Colao (116)
	0%	50%	1.5–3 mg/week CAB, 60-90 mg/month LAN	3 months	10	Marzullo (117)
	ns	44%	1.8 mg/week on average CAB, 30 mg/month OCT	8.44 months average	9	Gatta (118)
	ns	40%	1–10 mg three times daily BRC, 30 µg/month depot OCT	7 to 29 months	5	Selvarajah (119)
	ns	50%	1–1.5 mg/week CAB, 30 µg/month depot OCT	7 to 29 months	4	Selvarajah (119)
	ns	37%	2 or 3.5 mg/week CAB, 30 mg/month OCT	18 weeks	19	Mattar (120)
	0%	100%	30 mg/month OCT, 40 mg/day PEG	18 months	1	van der Lely (121)
	5%	58%	120 mg/month LAN, 40–80 mg/week or 40 or 60 mg twice/week PEG	7 months	57	van der Lely (122)
	0%	62%	median 30 mg/28 days OCT, median 15 mg/day PEG	9 months	29	Trainer (123)
	0%	56%	30 mg/28 days OCT, average 17.9 mg/day PEG	median 30 months	27	Bianchi (124)
	12%	97%	30 mg/28 days OCT or 120 mg/28 days LAN, median 80 mg/week PEG	median 59 months	112	Neggers (125)
	0%	100%	2.25 mg/week CAB, 60 mg/month PAS, 20 mg six times a week PEG	6 months	1	Ciresi (126)
ACTH	0%	67%	0.5 to 3mg/week CAB, 200–600 mg/day KCZ	12 months	6	Barbot (127)
	ns	67%	2–3 mg/week CAB, 200–400 mg/day KCZ	6 months	9	Vilar (128)
	ns	33%	0.5 mg every other day CAB, 250 µg three times daily PAS	33 days	12	Feelders (129)
	ns	75%	0.5 mg every other day CAB, 250 µg three times daily PAS, 200 mg three times daily KCZ	21 days	8	Feelders (129)

NFPA, non-functioning pituitary adenoma; ns, not stated; BRC, bromocriptine; TAM, tamoxifen; CAB, cabergoline; MET, metformin; OCT, octreotide; LAN, lanreotide; PEG, pegvisomant; KCZ, ketoconazole; PAS, pasireotide.

### DAs Based Therapies for Recurrent or Residual NFPAs

CAB monotherapy has been also used for recurrent or residual NFPAs (187 cases in six studies) (106–111). In these studies, in 33% of patients, CAB monotherapy resulted in more than 25% of tumor volume reduction or reduction of tumor size less than 2 mm in diameter, although the tumor reduction rate in these studies varies. The average dose of CAB was 2.95 mg/week and the average treatment duration was 8 months. The largest study, including 79 patients treated with DAs, demonstrated 35% reduction of tumor size on average when used against recurrent or residual NFPAs (111). It remains to be determined whether combinations of DAs with other drugs will be effective against refractory NFPAs.

### SSA Based Combination Therapies for SSA-Resistant Acromegaly

OCT resistance is estimated to occur in approximately 30% of GH-secreting adenomas and is thought to be mediated by reduced expression of SSTRs (130). It is found that three combination therapies (DA, pegvisomant or both) with SSA are mildly to moderately effective against these SSA-resistant GH-secreting tumors. As summarized in **Table 6**, these conventional combination therapies have shown a wide range of efficacies for controlling hormone levels (9%–100%) (112–126). The combination of PEG and SSA seems more effective than CAB and SSA to control IGF1 levels (112–126). None of the combinations are effective in reducing tumor size (0%–12%) (112–120).

### Combination Therapies for Aggressive Cushing Disease

Only a limited number of studies report treatment of aggressive Cushing disease with combinations of conventional therapies [**Table 6** (127–129)]. Ketoconazole (KCZ) in combination with CAB or PAS showed a moderate normalizing effect on cortisol levels in KCZ monotherapy resistant cases (129). CAB and PAS combinations were reported, but were less effective for reducing cortisol levels (129). One study showed no tumor reduction and the others did not mention efficacies on tumor size reductions.

## Epidermal Growth Factor Receptor (EGFR) Inhibitors

EGFR is a transmembrane receptor with the intracellular tyrosine kinase domain that regulates cell proliferation, migration, and survival. The EGFR inhibitors gefitinib and lapatinib are promising treatments for aggressive prolactinomas and aggressive corticotroph adenomas. EGFR signaling is detected in up to 82% of DA resistant prolactinomas. Activating EGFR signaling in rat lactosomatotroph cells causes elevation of *PRL* mRNA expression (131). EGFR signaling also upregulates the *POMC* (proopiomelanocortin) promoter and increases ACTH production in corticotrophs (132). Mutations in the ubiquitin specific peptidase 8 (*USP8*) gene, which causes gain-of-function of USP8 protein, are highly prevalent in Cushing disease (133). It is thought that the mutations in *USP8* cause enhanced EGFR signaling and are closely associated with development of Cushing disease (133–136).

Gefitinib has been implemented for treatment of both aggressive prolactinomas and corticotroph adenomas (137). Gefitinib suppressed PRL secretion by approximately 50% in human prolactinoma primary cultures *in vitro*. Moreover, gefitinib suppressed serum PRL levels by 40%–50% and tumor volume by 30% in somatotroph adenoma xenografted rodent models (131, 138). In primary cultures of ACTH-secreting human, canine, and murine adenomas, *POMC* mRNA was significantly suppressed by 63%–95% following treatment with gefitinib. In corticotroph adenoma xenografted mice, a 10-day course of treatment with gefitinib resulted in 40% inhibition of tumor growth (132).

Lapatinib is a dual EGFR inhibitor that inhibits both EGFR and human epidermal growth factor receptor 2 (HER2) (139). It has mostly been used to treat aggressive prolactinomas. A comparative study between lapatinib and gefitinib has found that lapatinib suppressed PRL levels more strongly in human prolactinoma primary cultures (60% with lapatinib and 40% with gefitinib) (138). It has also been reported that lapatinib suppresses PRL secretion (by 72%) and cell proliferation (by 80%) in EGFR and HER2-expressing transgenic mice, which are models of prolactinomas (140). In addition, a recent clinical trial treating two patients with DA-resistant prolactinomas with lapatinib at a dosage of 1,250 mg/day has reported 78% and 42% PRL suppression respectively with a 22% tumor reduction in the former and a stabilized tumor in the latter (28). These studies suggest that targeting EGFR signaling is a promising therapeutic strategy to, at least, populations of pituitary tumors with elevated EGFR signaling.

## Cyclin Dependent Kinase 2 (Cdk2) Inhibitor

CDK2 interacts with Cyclin E to facilitate entry into the S phase of the cell cycle and protects cells against apoptosis (141). The CDK2 inhibitor, roscovitine, has been tested in phase I and II clinical trials against various malignancies, including non-small cell lung cancer and nasopharyngeal and hepatocellular carcinomas (142). Roscovitine is currently being evaluated in preclinical studies for its ability to treat Cushing disease. Roscovitine targets the CDK2/Cyclin E complexes that are highly expressed specifically in corticotroph tumors (143). Roscovitine has dual effects; inhibition of corticotroph tumor growth and suppression of transcription of *POMC*, which encodes a precursor of ACTH (32, 143). It has been demonstrated that roscovitine can reduce *pomc* expression by over 50% in pituitary tumor transforming gene (PTTG) zebrafish model and reduced ACTH and corticosterone levels by 50% in corticotroph xenografted mice (143). In primary cells derived from human corticotroph tumors, roscovitine suppressed ACTH levels in five out of six tumors (83%) (32). These reports suggest promising results and may become a new avenue of treatment of Cushing disease.

## Retinoic Acid (RA)

RA is a vitamin A derivative that interacts with RA receptors and retinoid X receptors and regulates transcription of downstream genes (144, 145). In terms of the effects on pituitary hormone genes, studies have found that the all-trans and 9-cis isomers of RA reduce the DNA-binding affinity of NURR1, a nuclear orphan receptor important for the transcription of *POMC* (146). 9-cis RA has also been found to activate the transcription of *D2Rs*, suggesting that RA may be effective against both corticotroph adenomas and prolactinomas (147). As of the publication of this review, studies of the application of RA in the treatment of pituitary tumors have focused on the treatment of corticotroph adenomas.

The majority of studies utilizing RA are pre-clinical. It has been shown that RA treatment inhibits ACTH production and tumor growth in murine and canine models of Cushing disease (148, 149). Two other clinical studies have been published using RA to treat aggressive Cushing disease (33, 34). In one prospective study, urinary free cortisol levels normalized in 4 of 16 patients after treatment with RA (33). In a recent clinical trial, over 50% cortisol suppression was observed in five of seven recurrent pituitary tumors, with normalization in three patients (34). Of note, one patient who was resistant to CAB therapy had normalized urinary free cortisol levels following the addition of RA in the treatment (33). Moreover, a study utilizing primary culture derived from 11 corticotroph adenomas found that the combination of RA+BRC inhibited *POMC* transcription and ACTH production at higher levels than either alone in five of 11 cultures, indicating that RA+DA combination therapy may be a possible treatment for Cushing disease (33, 150). At this point, the use of RA remains at pre-clinical level, and further evidence is required to determine its efficacy.

## Conclusion

Aggressive pituitary tumors can be resistant to conventional therapies, including surgery, radiotherapy, and medical treatment. TMZ is recommended as the first-line chemotherapy for treatment of aggressive pituitary tumors. However, TMZ has a high rate of relapse in the long term. Several lines of evidence suggest the use of novel therapy, such as CAPTEM, PRRT, EVE, immunotherapy, EGFR inhibitors, CDK2 inhibitors, and RA, could be valuable strategies for long-term tumor control. These novel therapies could improve inhibition of pituitary tumor growth and/or control of excess hormone(s) compared to established treatment methods. Although information about the efficacy of such treatments is limited and few cases have utilized them so far, some treatments are showing promising outcomes. Further research will establish new treatment options and optimize treatment sequencing for aggressive pituitary tumors.

## Author Contributions

TA and TT designed the project. TN-T, KL, and JW did literature searches and analyzed data. TA, TT, TN-T, KL, JW, CM, and YK wrote the manuscript. All authors contributed to the article and approved the submitted version.

## Funding

This study is supported by grants from the Graduate School, University of Minnesota (UMF0011528 to TA, Grant-in-Aid 212588 to TA), the University of Alberta (Start-up to TT), the University Hospital Foundation (Medical Research Competition 2019 to TT), and the US National Institutes of Health (AR064195 to YK).

## Conflict of Interest

The authors declare that the research was conducted in the absence of any commercial or financial relationships that could be construed as a potential conflict of interest.
